# Research priority setting in obesity: a systematic review

**DOI:** 10.1007/s10389-021-01679-8

**Published:** 2021-12-03

**Authors:** Halima Iqbal, Rosemary R. C. McEachan, Jane West, Melanie Haith-Cooper

**Affiliations:** 1 Faculty of Health Studies, University of Bradford, Richmond Road, Bradford, BD7 1DP UK; 2 Bradford Institute for Health Research, Bradford Teaching Hospital NHS Foundation Trust, Bradford, UK

**Keywords:** obesity, research priority setting, obesity research agenda

## Abstract

**Aim:**

Obesity research priority setting, if conducted to a high standard, can help promote policy-relevant and efficient research. Therefore, there is a need to identify existing research priority setting studies conducted in the topic area of obesity and to determine the extent to which they followed good practice principles for research priority setting.

**Method:**

Studies examining research priority setting in obesity were identified through searching the MEDLINE, PBSC, CINAHL, PsycINFO databases and the grey literature. The nine common themes of good practice in research priority setting were used as a methodological framework to evaluate the processes of the included studies. These were context, use of a comprehensive approach, inclusiveness, information gathering, planning for implementation, criteria, methods for deciding on priorities, evaluation and transparency.

**Results:**

Thirteen articles reporting research prioritisation exercises conducted in different areas of obesity research were included. All studies reported engaging with various stakeholders such as policy makers, researchers and healthcare professionals. Public involvement was included in six studies. Methods of research prioritisation commonly included both Delphi and nominal group techniques and surveys. None of the 13 studies fulfilled all nine of the good practice criteria for research priority setting, with the most common limitations including not using a comprehensive approach and lack of inclusivity and evaluating on their processes.

**Conclusion:**

There is a need for research priority setting studies in obesity to involve the public and to evaluate their exercises to ensure they are of high quality.

## Introduction

Setting priorities for research helps to direct the most effective use of resources, such as research capacity, time and funds, to ensure an optimal health impact (Terry et al. 2018). Research priority setting in health, informed by stakeholders, can assist in the identification of topical and relevant issues, and unresolved questions regarding prevention, diagnosis and treatment of health conditions using a process that is explicit, iterative and inclusive (Rudan et al. 2010). There is currently no consensus on the definition of research priority setting, but there is agreement on a range of activities that centre on identifying, prioritising and reaching agreement on the research areas or questions deemed important to stakeholders (Tong et al. 2019). In the past, research-funding organisations and researchers developed their own research agendas without consulting key stakeholders (Graham et al. 2020). In recent times, however, there has been a focus on research needing to address questions that have relevance to those very people it intends to help (Dawson et al. 2017). It has been advocated that priority setting processes must also be fair, informed by credible evidence, of high quality and involve a broad range of stakeholders (Nasser et al. 2013; Sibbald et al. 2009; Viergever et al. 2010). Adopting a systematic and transparent approach to the identification of health research priorities can help to ensure that funded research has a public health benefit and make efficient and equitable use of limited resources (Bryant et al. 2014). Developing research agendas with target populations increases the potential for success and is more likely to be well received and relevant to their needs.

### Nine common themes of good practice in research priority setting

There are currently no published guidelines for reporting priority setting for health research (Tong et al. 2019). In the absence of a gold standard approach, a checklist of nine common themes for good practice in health research prioritisation was developed by Viergever et al. (2010). In developing the checklist, expert consultation was initiated, and a literature review identified several methodological approaches which were combined to draw together a comprehensive outline of common views on what constituted good practice in health research priority setting (Viergever and Roderik 2010). The aim was to facilitate a transparent and comprehensive priority setting via this checklist and accommodate the flexibility required by different contexts.

The nine themes contained within the checklist broadly fall into three different categories: *preparatory work, deciding on priorities* and *after priorities have been set.* Each category contains corresponding practices that further identify the goals in each step. There are five related practices within *preparatory work*, namely context, use of a comprehensive approach (established frameworks providing structured guidance for research prioritisation), inclusiveness, information gathering and planning for implementation. There are two related practices within *deciding on priorities,* namely criteria and methods for deciding on priorities, and two within *after priorities have been set,* namely evaluation and transparency. See Table 1 for a detailed description of each theme.

**Table 1:** Checklist for health research priority setting adapted from Viergever et al. (2010)

Theme	Description
Preparatory work	
1 - Context	1 The resources available for the exercise are reported. 2 The focus of the exercise is clearly stated, i.e. what it is about and who it is for). 3 The underlying values or principles are clear. 4 The health environment in which the process took place is described. 5 The research environment in which the process took place is described. 6 The political environment in which the process took place is described. 7 The economic/financial environment in which the process took place is described.
2 - Use of a comprehensive approach	8 The process of priority setting is described in detail.
3 - Inclusiveness	9 The participants involved in setting research priorities are described. 10 An appropriate representation of expertise is included. 11 An appropriate representation of the sexes is included. 12 An appropriate representation of regional participation is included. 13 Relevant health sectors and other constituencies are included.
4 - Information gathering	14 The information and sources used to inform the priority setting exercise are referenced.
5 - Planning for implementation	15 Plans for translation of research priorities are discussed. 16 Who will implement the research priorities and how?
Deciding on priorities	
6 - Criteria	17 Relevant criteria to focus discussion on setting priorities are stated.
7 - Methods for deciding on priorities	18 Approach for deciding on priorities is described (e.g. consensus or metrics based).
After priorities have been set	
8 - Evaluation	19 When and how evaluation of the established priorities and the priority setting process will take place is defined (e.g. multiple sessions).
9 -Transparency	20 Clarity about the approach used exists, i.e. how priorities are set.

The worldwide prevalence of obesity has significantly increased over the past few decades, leading the trend to be termed a ‘global epidemic’ by the World Health Organization and a serious threat to public health (World Health Organization 2017). Moreover, obesity is a global issue because it concerns both developed and developing countries (Cassi et al. 2017). The most recent available statistics from 2018/19 show that in England, a significant proportion of adults were overweight or obese, namely 67% of men and 60% of women (NHS Digital 2020). Of these, 26% of men and 29% of women were obese, and morbid obesity has also increased, from under 1% in 1993, to 3% in 2018 (NHS Digital 2020). Excess levels of fat in the body increase the risk of disease (Pollack et al. 2020) and obesity is a major risk factor for developing a range of conditions including cardiovascular disease, type 2 diabetes, muscular disorders, respiratory conditions and a host of psychological problems (Fruh 2017). A recent report by Public Health England highlights that the COVID-19 pandemic has brought to the fore the health crisis caused by overweight and obesity (Public Health England 2020). Both international and national research has consistently identified obesity as one of the key factors linked with severe outcomes from COVID-19 (Dietz and Santos-Burgoa 2020; Halvatsiotis et al. 2020). The direct annual costs resulting from obesity to the UK National Health Service (NHS) are reportedly estimated to reach £9.7 billion ($13.2 billion) by 2050, with wider costs to society predicted to reach just under £50 billion ($67.8 billion) per year by 2050 (Bradford Metropolitan District Council 2019).

Research is critical to inform prevention and treatment strategies to tackle obesity. Although there is a plethora of research examining the multitude of factors influencing obesity, research budgets are finite. Research priority setting can assist in making the most effective use of budgets by identifying the most relevant research areas according to different stakeholders. There is an emphasis on the need for research priority setting exercises to be explicit in their processes (Tong et al. 2019). Research priority setting guidelines and/or frameworks can help improve future research prioritisation in obesity, thus increasing the value and contribution of research aimed at reducing the obesity levels of populations.

### Objectives

The aim of this systematic review was to identify research priority setting exercises that have been conducted in obesity and to examine whether they had applied good practice principles in health research priority setting.

## Methods

The systematic review followed the standards of the Preferred Reporting Items for Systematic Reviews and Meta-Analyses (PRISMA) statement (Shamseer et al. 2015).

### Search strategy and process of study selection

The search was undertaken between 14–15 November 2020, using four electronic health databases, namely MEDLINE, PBSC, CINAHL and PsychINFO. The following Boolean search term combinations were used:

1. ‘research priority setting’ [all fields] OR ‘research prioritization’ [all fields] OR ‘research prioritisation’ [all fields] OR ‘research priorities’ [all fields] OR ‘research agenda’ [all fields]
  AND
2. ‘obesity’ OR ‘child obesity’ [all fields] OR ‘childhood obesity’ [all fields] OR ‘pediatric obesity’ [all fields] OR ‘obesity prevention’ [all fields] OR ‘obesity treatment’ [all fields]
3. We searched databases from their inception to November 2020. Only titles and abstracts published in English were included. The principal researcher (HI) independently conducted the article search. Searches in the grey literature included Google Scholar, Cochrane methods priority setting, the James Lind Alliance (a well-established priority-setting partnership method) and reference lists of selected articles to identify eligible papers. The search string ‘research priority setting and obesity’ was applied to Google Scholar. The first ten pages of Google Scholar were examined for additional articles. All authors contributed and refined the review’s search strategy.

### Inclusion and exclusion criteria

The review included any study describing a process of conducting a research prioritisation exercise in obesity. To be included in the review, studies must have outlined participants’ characteristics, stated the methods used to obtain research and identified well-established outcomes. International studies were included provided they were written in the English language. Studies were excluded if they did not mention health research, had not described the research prioritisation process or had assessed priorities for practice and policy rather than research (quality indicators). Also excluded were studies that did not focus on obesity research prioritisation.

**Fig. 1:**
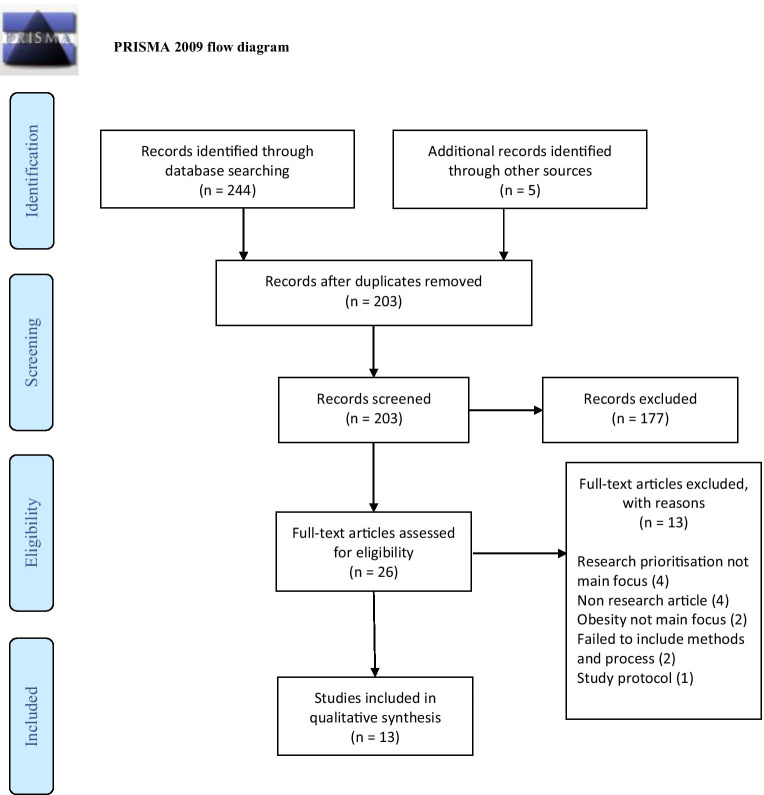
PRISMA 2009 flow diagram

Across all databases, the search yielded 249 citations, of which 203 remained after duplicates were removed. After the titles and abstracts had been screened, 26 articles underwent full-text screening. Of these publications, 13 studies met our inclusion criteria and were finally included in the analysis. Of the 13 excluded studies, four did not focus mainly on research prioritisation, one was a study protocol, two did not focus on obesity, four were non-research articles and two failed to include the methods and processes. All authors discussed and agreed on the selected papers. References were managed with EndNote X9 for ease. The PRISMA flowchart is displayed in Fig. 1.

### Quality assessment tool

In the absence of a gold standard approach to research priority setting, the checklist of nine common themes for good practice in health research priority setting by Viergever et al. (2010) was used to ascertain whether the research prioritisation exercises in each included study complied with good practice principles in their processes. This checklist has been previously used to evaluate or guide research prioritisation exercises (Doolan-Noble et al. 2019; Iqbal et al. 2021; Mador et al. 2016; Reveiz et al. 2013; Tong et al. 2015;) and has identified weaknesses prevalent in their processes. The checklist was specifically designed for health research priority setting and, as such, can identify issues that may have been otherwise overlooked by traditional quality appraisal tools.

### Data synthesis and extraction

A descriptive synthesis was conducted to outline study characteristics and outcomes, and to determine how many good practice principles each study followed. Studies could score between 0 (demonstrated none of the good practice principles) to 20 (demonstrated all of the good practice principles). One researcher (HI) independently extracted study characteristics, methods and outcomes. The relevant data were inserted into comprehensive data extraction checklist forms developed specifically for the quality synthesis. The quality appraisal criteria were applied by two researchers and resolved through discussion (HI and MC).

## Results

Studies were conducted in research priority setting in the area of obesity for childhood obesity (Botchwey et al. 2018; Byrne et al. 2008; Curtin et al. 2017; Gallagher et al. 2010; Hennessy et al. 2018; McPherson et al. 2016; Ramirez et al. 2011; Taylor et al. 2013; Ward et al. 2013), adult obesity (Hill et al. 2019; Hill et al. 2020; Mama et al. 2014), and obesity more generally (McKinnon et al. 2009). Studies were conducted in the areas of childhood obesity prevention or treatment (Byrne et al. 2008; Gallagher et al. 2010; Hennessy et al. 2018; Taylor et al. 2013), youth physical activity and healthy weight (Botchwey et al. 2018), healthy weight among youth with autism spectrum disorder and other developmental disabilities (Curtin et al. 2017), preconception priorities for maternal obesity prevention (Hill et al. 2019), pregnancy priorities for maternal obesity prevention (Hill et al. 2020), obesity reduction (Mama et al. 2014), obesity in children with physical disabilities (McPherson et al. 2016), obesity in Latino children (Ramirez et al. 2011), obesity policy (McKinnon et al. 2009) and obesity prevention in early care and education settings (Ward et al. 2013). The prioritisation exercises were all conducted in high income countries, namely Australia (4), the UK (1) and the US (8).

Seven studies did not include any patient or public involvement in their establishment of research priorities, yet involved a wide range of other stakeholders such as researchers, policy makers/leaders and healthcare professionals (Botchwey et al. 2018; Byrne et al. 2008; Gallagher et al. 2010; Hennessy et al. 2018; McKinnon et al. 2009; Taylor et al. 2013; Ward et al. 2013). One study solely involved the public in identifying priorities (Mama et al. 2014) and the remaining five studies involved the public alongside other stakeholders (Curtin et al. 2017; Hill et al. 2019; Hill et al. 2020; McPherson et al. 2016; Ramirez et al. 2011). Frequently cited methods used to identify priorities were surveys, Delphi techniques and the nominal group technique.

The main outcome of the studies was the generation of research priorities relevant to the topic and scope of each study. The priorities were described as prioritised research ideas/gaps/areas, prioritised lists, research priorities and prioritised themes. All 13 studies are displayed in Table 2 below.

**Table 2:** Study characteristics for the included empirical studies with quality scores

Study ID	Country	Topic and scope	Population included in the identification of priorities	Method	Main outcome (research priorities)	Quality score (based on met criteria in the checklist)
Botchwey et al. (2018)	USA	To develop a research agenda to address youth physical activity and healthy weight	Researchers from various disciplines. Health practitioners. No public involvement. Total n=unknown	Systematic literature reviews, online survey, discussions with practitioners and researchers	Research priorities were identified within various domains. Parks: How do different racial/ethnic groups use parks/trails to be physically active, especially children? Transportation, land use, urban design and community settings: How do play streets promote physical activity in elementary and middle school-aged kids, among different racial/ethnic groups living in lower-income rural communities? Out-of-school time: Which settings (hospitals, parks, etc.), provide the best opportunity to engage with and reach high-risk children in need of summer care?	12/20 (60%)
Byrne et al. (2008)	AUS	To identify priorities for longitudinal research in child obesity	Researchers, medical practitioners, dietitians, scientists and other healthcare professionals interested in obesity research, treatment or public health initiatives directed at the prevention of obesity. No public involvement. (Total n=71)	Two-stage Delphi	Research questions were identified and ranked in order of importance. The highest-priority questions related to modifiable environmental risk/protective factors; parental and family factors; longitudinal relationships between the development of obesity and physical, social and mental health; predisposing prenatal and early childhood patterns of growth and nutrition; identification of stronger early markers of chronic disease risk in later years, and better understanding of the natural course of overweight in childhood	8/20 (40%)
Curtin et al. (2017)	USA	To develop a research agenda to address obesity in children with autism and developmental disabilities	Researchers, family members, self-advocates and policy makers. (Total n=38)	Three-round modified Delphi	The five research areas identified for priority were: (1) family practices around food/mealtimes; (2) physical activity and sedentary behaviours in relation to weight; (3) relationship between food patterns, behaviour, and weight gain; (4) programme-adaption and delivery; and (5) influence of school and community-based organizations on food intake and physical activity	12/20 (60%)
Gallagher et al. (2010)	USA	To identify interdisciplinary research priorities for pediatric obesity prevention and treatment	University faculties from dentistry, education, medicine, nursing, nutrition, pediatrics, psychology, public health, and social work. No public involvement. (Total n=55)	Workshops. Focus groups	Top 10 prioritised areas included: (1) integration of behavioural and cultural components into research; (2) contribution of health disparities on rates of childhood obesity in our communities; (3) social determinants of health and identification of previously unmeasured factors; (4) effectiveness of behavioural approaches targeted towards families; and (5) translating current evidence into practice in the clinic and community	12/20 (60%)
Hennessy et al. (2018)	UK	To identify research priorities in childhood obesity prevention	Researchers, policy makers, and healthcare practitioners. No public involvement. (Total n=77)	Two rounds of nominal group technique	The top five research priorities identified were: (1) evaluation (including economic evaluation), current programmes to inform practice and policy; (2) how to change culture towards addressing the determinants of health; (3) implementation science: investigate process; (4) how to integrate obesity prevention into existing service structures; and (5) how to enhance opportunities for habitual physical activity, including free play and active travel	10/20 (50%)
Hill et al. (2019)	AUS	To generate preconception research priorities for maternal obesity prevention	Researchers, clinical stakeholders, academics, public representatives*. (Total n=21). *Public stakeholders constituted 10% of total sample	Systematic review, three rounds of modified Delphi, and nominal group technique	Five preconception research priorities and four overarching principles were identified. The priorities were: (1) healthy diet and nutrition; (2) weight management; (3) physical activity; (4) planned pregnancy; and (5) physical, mental and psychosocial health	14/20 (70%)
Hill et al. (2020)	AUS	To generate pregnancy research priorities to address rising maternal obesity	Researchers, public representatives*. (total n=20) *Public stakeholders constituted 10% of total sample	Modified Delphi and nominal group technique	Research priorities identified included optimising: (1) healthy diet and nutrition; (2) gestational weight management; (3) screening for and managing pregnancy complications and pre-existing conditions; (4) physical activity; (5) mental health; and (6) postpartum (including intrapartum) care	13/20 (65%)
Mama et al. (2014)	USA	To explore and describe community perceptions of the causes of obesity and possible solutions to inform a collaborative research agenda	Community members representing a wide range of occupations including healthcare practitioners and technicians, financial and business, and community and social services. (Total n=22)	Interviews.	Common problems identified were: (1) childhood obesity; (2) balancing a healthy diet and physical activity. Additional emergent themes focused on solutions, including increasing awareness and education, coordinated efforts among organizations, and using an ecologic approach to combat obesity	11/20 (55%)
McPherson et al. (2016)	USA	To identify the obesity needs of children with physical disabilities to inform future research	Researchers, trainees, front line clinicians, decision makers, parents*, former clients with disabilities*, community partners*. (Total n=38). *Public stakeholders constituted 7% of total sample	Modified nominal group technique	Three high-priority areas were: (1) early, sustained engagement of families; (2) rethinking determinants of obesity and health; and (3) evidence-informed measurement and outcomes	14/20 (70%)
Ramirez et al. (2011)	USA	To identify research priorities to address Latino childhood obesity	Academics, researchers, health educators, administrators, managers, clinicians, public health workers, students, community*. (Total n=313). *Public stakeholders constituted 0.6% of total sample	Modified three-round Web-based Delphi	Twenty-five research priorities identified within the domains of society, community, school, family and individual. These included policies that subsidize accessibility of healthy foods to improve diet among Latino families, built environment policies involving collaborations with multiple stakeholders to promote physical activity, school health, nutrition and active physical education classes as part of the school curriculum, and engaging Latino families as advocates of childhood obesity prevention initiatives at the community and school levels. Individual: programs making physical activity more attractive than watching TV or playing video games	14/20 (70%)
McKinnon et al. (2009)	USA	To identify priorities for a research agenda to inform obesity policy	Experts in medicine, public health, nutrition, physical activity, economics, health policy and legislation, and healthcare delivery systems. No public involvement. (Total n=27)	Semi-structured telephone interviews. Modified nominal group process	Themes that emerged were: (1) the embryonic nature of obesity policy research; (2) the need to study ‘natural experiments’ resulting from policy-based efforts to address the obesity epidemic; (3) the importance of research focused beyond individual-level behaviour change; (4) the need for economic research across several relevant policy areas; and (5) the overall urgency of taking action in the policy arena	10/20 (50%)
Taylor et al. (2013)	AUS	To determine which research topics are considered most important for the effective management of obesity in children	Researchers. No public involvement. (Total n=78)	Three-round Delphi	The highest research priorities identified were: (1) determining the best strategies for long-term weight management; and (2) identifying how best to support the primary healthcare system to achieve these strategies	6/20 (30%)
Ward et al. (2013)	USA	To develop priorities for future research on healthy weight development in children aged 2–5 years	Researchers, investigators, and leaders in early care and education. No public involvement. (Total n=43)	Meeting Survey.	Highest-rated issues included: (1) assessment of the quality of children’s meals and snacks; (2) use of financial incentives; (3) interventions that involve healthcare providers (4); the role of screen time; and (5) the need for multilevel interventions	10/20 (50%)
Countries key						
USA			United States of America			
AUS			Australia			
UK			United Kingdom			

When matched against the checklist of good practice principles in research priority setting as defined by Viergever et al. (2010), none of the studies adhered to all the principles outlined in the checklist (see Table 3).

**Table 3:** Appraisal of comprehensiveness of reporting

Item	Studies that fulfilled the principles outlined in the checklist	Total studies *(out of a total of 13)*
Context		
*1 - The resources available for the exercise are reported*	Curtin et al. (2017); Gallagher et al. (2010); Hennessy et al. (2018); Hill et al. (2020); Hill et al. (2019); Mama et al. (2014); McKinnon et al. (2009); McPherson et al. (2016); Ward et al. (2013)	9
*2 - The focus of the exercise is clearly stated, i.e. what it is about and who it was for*	Botchwey et al. (2018); Byrne et al. (2008); Curtin et al. (2017); Gallagher et al. (2010); Hennessy et al. (2018); Hill et al. (2020); Hill et al. (2019); Mama et al. (2014); McKinnon et al. (2009); McPherson et al. (2016); Taylor et al. (2013); Ramirez et al. (2011); Ward et al. (2013)	13
*3 - The underlying values or principles are clear*	Botchwey et al. (2018); Byrne et al. (2008); Curtin et al. (2017); Gallagher et al. (2010); Hennessy et al. (2018); Hill et al. (2020); Hill et al. (2019); Mama et al. (2014); McKinnon et al. (2009); McPherson et al. (2016); Ramirez et al. (2011); Taylor et al. (2013); Ward et al. (2013)	13
*4 - The health environment in which the process took place is described*	Botchwey et al. (2018); Curtin et al. (2017); Gallagher et al. (2010); Hennessy et al. (2018); Hill et al. (2020); Hill et al. (2019); Mama et al. (2014); McKinnon et al. (2009); McPherson et al. (2016); Ramirez et al. (2011); Ward et al. (2013)	11
*5 - The research environment in which the process took place is described*	Byrne et al. (2008); Curtin et al. (2017); Gallagher et al. (2010); Hennessy et al. (2018); Hill et al. (2020); Hill et al. (2019); Mama et al. (2014); McPherson et al. (2016); McKinnon et al. (2009); Ramirez et al. (2011); Taylor et al. (2013); Ward et al. (2013)	12
*6 - The political environment in which the process took place is described*	0	0
*7 - The economic/financial environment in which the process took place is described*	0	0
Use of a comprehensive approach		
*8 - The process of priority setting is described in detail*	0	0
Inclusiveness		
*9 - The participants involved in setting research priorities are described*	Botchwey et al. (2018); Byrne et al. (2008); Curtin et al. (2017); Gallagher et al. (2010); Hennessy et al. (2018); Hill et al. (2020); Hill et al. (2019); Mama et al. (2014); McKinnon et al. (2009); McPherson et al. (2016); Ramirez et al. (2011); Taylor et al. (2013); Ward et al. (2013)	13
*10 - An appropriate representation of expertise is included*	Curtin et al. (2017); Hill et al. (2020); Hill et al. (2019); Mama et al. (2014); McPherson et al. (2016); Ramirez et al. (2011)	6
*11 - An appropriate representation of the sexes is included*	Mama et al. (2014)	1
*12 - An appropriate representation of regional participation is included*	Botchwey et al. (2018); Byrne et al. (2008); Curtin et al. (2017); Gallagher et al. (2010); Hennessy et al. (2018); Hill et al. (2020); Hill et al. (2019); Mama et al. (2014); McPherson et al. (2016); Ramirez et al. (2011)	10
*13 - Relevant health sectors and other constituencies are included*	Botchwey et al. (2018); Byrne et al. (2008); Curtin et al. (2017); Hill et al. (2019); McKinnon et al. (2009); McPherson et al. (2016); Ramirez et al. (2011); Ward et al. (2013)	8
Information gathering		
*14 - The information and sources used to inform the priority setting exercise are referenced*	Botchwey et al. (2018); Byrne et al. (2008); Curtin et al. (2017); Gallagher et al. (2010); Hennessy et al. (2018); Hill et al. (2020); Hill et al. (2019); Mama et al. (2014); McKinnon et al. (2009); McPherson et al. (2016); Ramirez et al. (2011); Taylor et al. (2013); Ward et al. (2013)	13
Planning for implementation		
*15 - Plans for translation of research priorities are discussed*	Botchwey et al. (2018); Gallagher et al. (2010); Hill et al. (2020); Hill et al. (2019); McPherson et al. (2016); Ramirez et al. (2011)	6
*16 - Who has implemented the research priorities and how?*	Botchwey et al. (2018); Gallagher et al. (2010); Hill et al. (2020); Ramirez et al. (2011)	4
Criteria		
*17 - Relevant criteria to focus discussion on setting priorities are stated*	Botchwey et al. (2018); Hill et al. (2020); Hill et al. (2019); McKinnon et al. (2009); McPherson et al. (2016); Ramirez et al. (2011)	6
Methods for deciding on priorities		
*18 - Approach for deciding on priorities is described (e.g. consensus or metrics based)*	Botchwey et al. (2018); Byrne et al. (2008); Curtin et al. (2017); Gallagher et al. (2010); Hennessy et al. (2018); Hill et al. (2020); Hill et al. (2019); Mama et al. (2014); McKinnon et al. (2009); McPherson et al. (2016); Ramirez et al. (2011); Taylor et al. (2013); Ward et al. (2013)	13
Evaluation		
*19 - When and how evaluation of the established priorities and the priority setting process will take place is defined (e.g. multiple sessions)*	0	0
Transparency		
*20 - Clarity about the approach used exists, i.e. how priorities are set*	Botchwey et al. (2018); Byrne et al. (2008); Curtin et al. (2017); Gallagher et al. (2010); Hennessy et al. (2018); Hill et al. (2020); Hill et al. (2019); Mama et al. (2014); McPherson et al. (2016); Ramirez et al. (2011); Taylor et al. (2013); Ward et al. (2013)	12

### Summary of the comprehensiveness of studies in reporting good practice principles

#### Theme 1: Context

The focus of the exercise was made clear in all studies, as were the underlying values and principles of each study. These included the need to engage the community in identifying obesity research priorities (Mama et al. 2014), or to foster collaboration amongst interdisciplinary research experts in the field of healthy weight, prevention of weight gain and maintenance of healthy weight (Gallagher et al. 2010; Hennessy et al. 2018; Taylor et al. 2013), or to develop a research agenda leveraging the collective expertise of a range of stakeholders (McPherson et al. 2016). However, the resources used for the exercises were made explicit in very few studies. Where information was provided, these included the use of materials used during the exercise such as cards to write knowledge gaps on (McPherson et al. 2016), flipcharts and numbered stickers for ranking (Hennessy et al. 2018), the use of audio-recorders (Mama et al. 2014) and the use of facilitators (Gallagher et al. 2010; Hennessy et al. 2018; Hill et al. 2020; Hill et al. 2019; McKinnon et al. 2009; McPherson et al. 2016) and project staff members to take notes and capture details around the issues raised (Ward et al. 2013), as well as the use of a statistician, data analyst and administrative support staff (Curtin et al. 2017). In one study, the use of a transcription service was disclosed (Mama et al. 2014). The economic/financial and political environment in which the prioritisation exercise took place was not disclosed in any of the studies.

#### Theme 2: Use of a comprehensive approach

None of the studies reported the use of established, structured, step-by-step frameworks specifically designed for research priority setting to guide their prioritisation processes, such as the James Lind Alliance (JLA) methodology (JLA 2020), the Essential National Health Research (ENHR) strategy (COHRED 2009), the Combined Approach Matrix (CAM) (Ghaffar 2009) and the Child Health and Nutrition Research Initiative (CHNRI) (Rudan 2016). None of the studies developed their own frameworks to guide their exercises.

#### Theme 3: Inclusiveness

Across prioritisation exercises, participants comprised a diverse range of stakeholders. Samples were inclusive of health service managers, medical practitioners, healthcare practitioners, academics, interdisciplinary researchers, dietitians, scientists, government agencies, policy leaders and experts in the field of child obesity more generally. Two studies solely involved researchers in the process (Gallagher et al. 2010; Taylor et al. 2013). Public involvement in the exercise was made explicit in six studies only (Curtin et al. 2017; Hill et al. 2020; Hill et al. 2019; Mama et al. 2014; McPherson et al. 2016; Ramirez et al. 2011). Although all studies discussed participant characteristics, some were more detailed in their descriptions by disclosing the sex of participants (Hennessy et al. 2018; Mama et al. 2014; Ramirez et al. 2011), with women overwhelmingly outnumbering men in two studies (Hennessy et al. 2018; Ramirez et al. 2011). An appropriate representation of regional participation was included in most studies that did not involve the public, as well as the incorporation of relevant sectors.

#### Theme 4: Information gathering

In some studies, a core planning group or committee suggested initial priorities to direct the process (Gallagher et al. 2010; Ramirez et al. 2011; Ward et al. 2013), or researchers identified the initial areas and other stakeholders prioritised the selected areas (Botchwey et al. 2018; Byrne et al. 2008). The use of technical data was reported in most studies. These included reviews of guidelines and recommendations (Hill et al. 2020; Hill et al. 2019), as well as literature searches, reports and systematic reviews (Botchwey et al. 2018; Hill et al. 2020; Ramirez et al. 2011). Surveys were conducted to obtain broad input on the selected topic areas (Botchwey et al. 2018; Byrne et al. 2008; Curtin et al. 2017), as were questionnaires (Ramirez et al. 2011; Taylor et al. 2013). Workshops (Gallagher et al. 2010; Hennessy et al. 2018; Hill et al. 2019; Hill et al. 2020; McPherson et al. 2016), group meetings (Curtin et al. 2017; McPherson et al. 2016; Ward et al. 2013) and brainstorming sessions were also reported as a means of generating information (Curtin et al. 2017), as well as presentations (McPherson et al. 2016; Ward et al. 2013).

#### Theme 5: Planning for implementation

Most of the studies did not report their plans for implementing identified priorities. Several community projects were established from two research priority setting studies (Gallagher et al. 2010; Ramirez et al. 2011). Plans for implementing pilot studies were established from a research agenda (Ramirez et al. 2011). Ongoing activities influenced by the identified priorities were reported in two studies (Hill et al. 2019; Hill et al. 2020). The research agenda shaped four initial projects in another study (Botchwey et al. 2018) and finally, one study secured a large team grant to address some items on their research agenda (McPherson et al. 2016).

#### Theme 6: Criteria

Criteria to focus discussion on research priorities were mentioned in six studies (Botchwey et al. 2018; Hill et al. 2020; Hill et al. 2019; McKinnon et al. 2009; McPherson et al. 2016; Ramirez et al. 2011). Cited criterion included research priorities that had the greatest long-term impact, and what would have the most immediate impact (Botchwey et al. 2018), prevalence or burden attributable to the proposed problem (Hill et al. 2019), provision, potential and proposed transformation attributable to the problem (Hill et al. 2020), preventative effect with respect to obesity development, and implementation feasibility (Hill et al. 2020), and the most appropriate and feasible methods for initiating research efforts (McPherson et al. 2016).

#### Theme 7: Methods for deciding on priorities

Studies either adopted a metrics approach (Botchwey et al. 2018; Byrne et al. 2008; Curtin et al. 2017; Gallagher et al. 2010; Taylor et al. 2013; Ward et al. 2013), a consensus approach (McPherson et al. 2016; Ramirez et al. 2011) or a combination of both (Hennessy et al. 2018; Hill et al. 2019; Hill et al. 2020). Likert scales were utilised in one study for ranking priorities (Ramirez et al. 2011), as were numbered stickers (Hennessy et al. 2018). The Delphi method was the most used method for deciding on priorities, both in its original form (Byrne et al. 2008; Ramirez et al. 2011; Taylor et al. 2013) and adapted versions, followed by the nominal group technique (Hennessy et al. 2018). In two studies, the Delphi technique was combined with the nominal group technique (Hill et al. 2019; Hill et al. 2020). One study used a modified nominal group technique to determine priorities (McKinnon et al. 2009). Another study did not use ranking and/or consensus to determine priorities, and instead searched for themes in the data and described these as the priorities (Mama et al. 2014).

#### Theme 8: Evaluation

There were no reported plans to update the priorities. One study mentioned that the research agenda would be reviewed, re-evaluated and refined (Curtin et al. 2017).

#### Theme 9: Transparency

Most of the studies were explicit in their priority setting processes, despite not using a well-established framework, although some were more transparent than others (Gallagher et al. 2010; Hennessy et al. 2018; Hill et al. 2020; Hill et al. 2019; Ramirez et al. 2011). The majority of studies outlined how the priorities were set. In most cases, it was clear which stakeholders identified initial topics, which stakeholders added generated additional input and who exactly prioritised.

Some studies also highlighted the limitations of their prioritisation exercise, such as acknowledging the lack of public involvement altogether (Hennessy et al. 2018), the possibility of unequal representation of disciplines (Hill et al. 2019; Hill et al. 2020), the lack of participation in person by children or youth (McPherson et al. 2016) and the lack of men that participated (Hennessy et al. 2018). Further highlighted limitations were around the issue of generalisability. This included the small sample size (Taylor et al. 2013), method of sample recruiting (Mama et al. 2014) and the possibility of selection bias due to the participants not being randomly selected (Ramirez et al. 2011). Other challenges were also highlighted, such as issues encountered in achieving consensus during the prioritisation phases (Hennessy et al. 2018), and the steps taken to reduce potential limitations when using the nominal group technique (Hennessy et al. 2018; Hill et al. 2019). One study reported pilot testing the questionnaire used to elicit priorities utilising a survey instrument, and subsequently revising it for improvement (Ramirez et al. 2011).

## Discussion

This review provides an assessment of research priority setting initiatives in the area of obesity. Most of the prioritisation exercises focussed on obesity topics including causes, prevention and management. Of the 13 identified studies, ten concentrated on child obesity, three on adult obesity and one focussed on obesity more generally. The application of a checklist of good practice principles in research priority setting identified the strengths and weaknesses inherent in each study. None of the studies fulfilled all the good practice principles as outlined by the checklist. It is clear that more effort needs to be made in studies examining obesity research priority setting to ensure that their processes are of a high quality. It is important to note however, that two studies (Byrne et al. 2008; McKinnon et al. 2009) were conducted before the checklist of nine common themes of good practice was published in 2010. In addition, literature advocating the need for research priority setting to be fair, legitimate, informed by credible evidence, include a wide range of stakeholders and be transparent, has only more recently been strongly advocated (Bhaumik et al. 2015; Nasser et al. 2013; Tong et al. 2019; Viergever et al. 2010) which may be as a result of the increase in research prioritisation exercises in the past two decades. Our findings suggest that the greatest limitations of studies when applied to the checklist of good practice concerned the criteria use of comprehensive approach, inclusiveness and evaluation.

None of the studies used comprehensive well-established research priority setting frameworks such as the JLA methodology, the ENHR strategy, the CAM and the CHNRI initiative. These established schemata were all developed before the studies were undertaken and provide step-by-step guidance for the entire process, while covering many of the points on the checklist (Viergever et al. 2010). It is argued by Viergever et al. (2010) that the use of these structurally well-defined tools and methods should at least be considered, and that they will gradually replace commonly used methods such as the Delphi method (Yoshida 2016), which was a frequently used method used to establish obesity priorities in the identified studies.

It is concerning that only six of the 13 studies in this review involved the public as stakeholders and even then, the public were significantly underrepresented in the sample (Hill et al. 2020; Hill et al. 2019; McPherson et al. 2016; Ramirez et al. 2011), with another study not making clear how many public stakeholders were involved in the process (Curtin et al. 2017). Interestingly, of the seven studies that scored the highest in this review, six of them involved the public in the generation of priorities. It is well established in the literature that community engagement in research priority setting is crucial for establishing research questions that are relevant to them. Previous studies have demonstrated that the research priorities of other stakeholders do not align with those of the public (Brady et al. 2020; Manikam et al. 2017; Owens et al. 2008; Tallon et al. 2000; Voigt et al. 2010). A 2014 report systematically reviewed research priority setting studies from the period 1966 to 2014 and found that in the 91 studies, researcher and government involvement was strong, yet involvement of other key stakeholders was limited (McGregor et al. 2014). To ensure the incorporation of public and patients in the process, guidelines are available such as the Guidance for Reporting Involvement of Patients and the Public (GRIPP) checklist (Staniszewska et al. 2017), which was developed to aid in improving the quality, consistency and transparency of reporting the inclusion of patients and the public in research. The checklist offers a comprehensive list of issues that require consideration when reporting activities in relation to public and patient involvement. It must be noted, however, that it fails to offer information on how the public and patient contributors are to be recruited (Dawson et al. 2017). Additionally, it does not offer explicit consideration for representing the diversity of the population relevant to the topic area (Dawson et al. 2017). It is unclear in the current review whether public stakeholders were representative of the community at large, i.e. whether there was inclusion of Black and minority ethnic stakeholders in the samples. In addition to ensuring the inclusion of the public in research priority setting exercises, it is recommended that key characteristics of the sample are recorded and reported so that issues in relation to inclusion and diversity can be understood.

With regard to evaluation, a small number of studies in this review described strategies for the implementation of identified priorities, yet none measured the impact of the prioritisation. This can be done, for example, by performing an impact assessment reviewing the research performed (Viergever and Roderik 2010). The authors of a 2014 report (McGregor et al. 2014) argued that many of the exercises failed to translate the result of the prioritisation process into implementation of projects. It was further highlighted that the exercises were rarely repeated due to the lack of follow-up. The authors of the current review would strongly endorse the use of good practice guidelines, such as the one used to critically appraise the studies in this review, or the Reporting Guideline for Priority Setting of Health Research (REPRISE) by Tong et al. (2019).

## Conclusion

In summary, one can say that while research priority setting studies in the topic area of obesity do exist, they vary in scope and in quality. Although a wide range of stakeholders were involved in the prioritisation processes, public involvement was either non-existent or limited. The use of a comprehensive approach in research priority setting and/or adherence to good practice guidelines could enrich obesity priority setting processes to ensure the identified obesity priorities are relevant, transparent and can assist in implementation efforts. It is imperative that the public be involved in the obesity research priority setting process, resulting in research agendas that have incorporated their unmet needs. This can improve the relevance and legitimacy of research and ultimately achieve better health outcomes in obesity.

## Data Availability

DOI’s are cited in the reference list
